# A case report of painless type A aortic dissection with intermittent convulsive syncope as initial presentation

**DOI:** 10.1097/MD.0000000000006762

**Published:** 2017-04-28

**Authors:** Chun-Hsien Chen, Kuan-Ting Liu

**Affiliations:** a Department of Emergency Medicine, Kaohsiung Medical University Hospital; b School of Medicine, College of Medicine, Kaohsiung Medical University, Kaohsiung, Taiwan.

**Keywords:** cardiac asystole, convulsive syncope, painless type A aortic dissection

## Abstract

**Rationale:**

The initial presenting symptoms and signs of acute aortic dissection are so diverse that it makes early and accurate diagnosis arduous. Painless and convulsive syncope due to cardiac arrhythmia were not typical presentations of acute aortic dissection.

**Patient concerns:**

A 61-year-old male presenting with transient consciousness loss and suspected seizure attack was sent to emergency room (ER) by ambulance. Consciousness loss accompanying with upward gaze and limb convulsion was noted in ER, and electrocardiogram monitor recorded a transient cardiac asystole then spontaneous recovery of sinus rhythm.

**Diagnoses:**

Chest X-ray revealed widening of the mediastinum. Subsequently, contrast-enhanced chest computed tomography demonstrated Stanford type A aortic dissection.

**Lessons:**

To the authors’ knowledge, this is the first reported case that cardiac asystole may be related to painless type A aortic dissection and then leading to convulsive syncope as presenting symptoms.

## 1 Introduction

Acute aortic dissection is not uncommon but a challenge disease for emergency physicians, and it may be catastrophic if it not be diagnosed and managed promptly. The initial presenting symptoms and signs of acute aortic dissection are so diverse that it makes early and accurate diagnosis arduous. The Stanford classification is divided into two groups, A and B, depending on whether the ascending aorta is involved. The Stanford type A aortic dissection involves the ascending aorta and/or aortic arch and has higher mortality and generally requires primary surgical treatment.^[1]^ Here, we presented a case of Stanford type A aortic dissention with intermittent transient consciousness loss and convulsive movement but no chest or back pain as initial manifestation that may be consequences of transient cardiac asystole caused by aortic dissection. The ethical approval was not necessary for this case report article under the regulations of institutional review board of the Kaohsiung Medical University Hospital.

## 2 Case report

**Figure 1:**
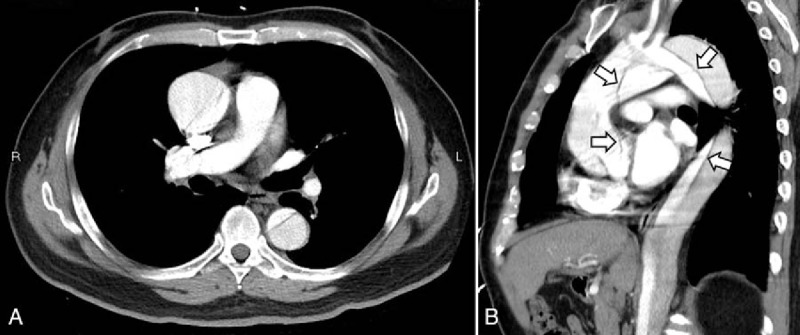
Contrast-enhanced computed tomography of chest indicated type A aortic dissection from aortic root. (A) Axial view; (B) sagittal view. Arrows indicate intimal flap.

**Figure 2:**
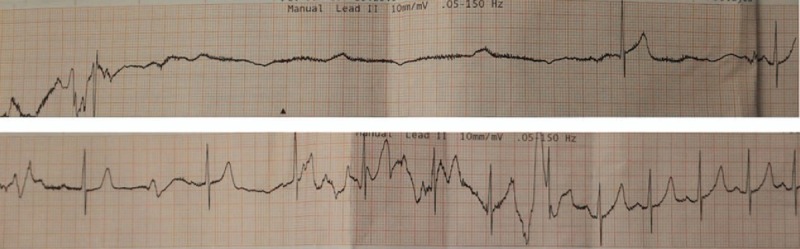
Transient cardiac asystole then spontaneous recovery of sinus rhythm recorded on electrocardiogram monitor while syncope convulsion.

A 61-year-old male with history of hypertension presenting with transient consciousness disturbance and suspected seizure attack was sent to emergency room (ER) by ambulance. At arrival, his was clear, afebrile, no tachypnea, no tachycardia, no hypotension (blood pressure [BP]: 115/59 mm Hg), and no hypoxia (SpO_2_: 100%). He reported a sensation of out of breath and then loss of consciousness. He denied headache, chest pain, back pain, and abdomen pain. Initial 12-lead electrocardiogram (EKG) demonstrated normal sinus rhythm. Loss of consciousness with convulsive movement was noted in the ER while we were waiting for laboratory tests results. Therefore, he was transferred to critical area and on EKG monitor, BP monitor, and oximeter. His hemogram indicated mild anemia (hemoglobin: 11.8 mg/dL and hematocrit: 35.1%). Liver function test and creatinine level were unremarkable, but there were increased C-reactive protein (122 mg/L) and d-dimer (1.56 mg/L) level. Blood gas analysis was also unremarkable. Computed tomography (CT) of head without contrast enhancement did not reveal ischemic changes, intracranial hemorrhages, or space-occupying lesions. However, his chest radiography indicated a widening of mediastinum, and contrast-enhanced chest CT demonstrated Stanford type A aortic dissection extending from the aortic root to the bifurcation of common iliac arteries (Fig. 1). Consciousness loss accompanying with upward gaze and limb convulsion for about 10 seconds was noted again in ER, and EKG monitor recorded a transient cardiac asystole then spontaneous recovery of sinus rhythm without cardiopulmonary resuscitation (Fig. 2). The patient underwent a successful operative replacement of the ascending aorta and semiarch replacement with a Dacron graft as well as aortic valve suspension.

## 3 Discussion

Atypical presentations of aortic dissection make promptly accurate diagnosis difficult, especially in painless aortic dissections. Cardiac arrhythmias have been reported in some case reports; however, to the authors’ knowledge, there is no cardiac systole reported in the scenario of painless type A aortic dissection.

Abrupt onset pain was the most common presenting symptom in the International Registry of Acute Aortic Dissection study.^[2]^ Approximate 80% of patient complained of chest pain, and anterior chest pain was in majority. Pain is not an obligatory symptom of acute aortic dissection, though painless acute aortic dissections were relatively uncommon (6.4%). It is intriguing that type A aortic dissection was more frequent in painless group.^[3]^ Syncope, congestive heart failure, and stroke were more frequent presenting signs in painless dissection patients. In-hospital mortality of painless aortic dissections was higher especially due to type B dissection. In one retrospective study, neurologic symptoms as initial presenting manifestation of aortic dissection account for approximate 30% of patients.^[4]^ Neurological symptoms were including ischemic stroke (16%), ischemic neuropathy (11%), syncope (6%), somnolence (4%), and seizures (3%).^[5]^ Convulsive syncope has been reported to be associated with episodes of cardiac asystole documented by implantable loop recorder and may be corrected by pacemaker implantation.^[6]^

The majority (71.4%) of painless aortic dissections had normal EKG, and approximate 11.4% of cases showed cardiac arrhythmias in a retrospective study.^[7]^ Aortic dissection associated with different arrhythmias such as atrioventricular block, atrial fibrillation,^[8]^ supraventricular tachycardia,^[9]^ and bigeminal premature ventricular contractions^[10]^ had been reported. Complete heart block as a presenting sign of painless type A aortic dissection was also documented.^[11]^ However, a painless type A aortic dissection associated with transient cardiac asystole and spontaneous recovery in our case report may be the first one in the literature.

Dynamic obstruction occurs as a result of occlusion of the suppressed true lumen of the aorta by the increased and pressurized false lumen. In one case report,^[12]^ the patient with type B aortic dissection had cardiac arrest in which acute dynamic obstruction was regarded as a cause. Coronary malperfusion may be a cause of cardiac arrest. Type A aortic dissection may cause coronary malperfusion via mechanical obstruction in coronary ostia without dissection extending in the coronary arteries.^[13]^ In the present case, aortic dissection-related dynamic obstruction may lead to coronary malperfusion and then cause cardiac arrest.

In summary, the case we reported here is that patient had recurrent convulsive syncope episodes as initial presenting manifestation of painless acute type A aortic dissection, and we recorded a transient cardiac asystole on EKG monitor while he suffered from convulsive syncope in the ER. Cardiac asystole may be caused by coronary malperfusion via dynamic obstruction of aortic root dissection and then cerebral perfusion was impeded, therefore, may induce convulsive syncope of the patient.
